# Correction of lower limb deformity in an adult patient with Ellis-van Creveld syndrome: a rare case report

**DOI:** 10.1093/jscr/rjae648

**Published:** 2024-10-11

**Authors:** Yukie Metoki, Dai Iwase, Ryo Ota, Jun Aikawa, Manabu Mukai, Kensuke Fukushima, Gen Inoue, Masashi Takaso

**Affiliations:** Department of Orthopedic Surgery, Kitasato University School of Medicine, 1-15-1 Kitasato, Minami-ku, Sagamihara, Kanagawa 252-0374, Japan; Department of Orthopedic Surgery, Kitasato University School of Medicine, 1-15-1 Kitasato, Minami-ku, Sagamihara, Kanagawa 252-0374, Japan; Department of Orthopedic Surgery, Kitasato University School of Medicine, 1-15-1 Kitasato, Minami-ku, Sagamihara, Kanagawa 252-0374, Japan; Department of Orthopedic Surgery, Kitasato University School of Medicine, 1-15-1 Kitasato, Minami-ku, Sagamihara, Kanagawa 252-0374, Japan; Department of Orthopedic Surgery, Kitasato University School of Medicine, 1-15-1 Kitasato, Minami-ku, Sagamihara, Kanagawa 252-0374, Japan; Department of Orthopedic Surgery, Kitasato University School of Medicine, 1-15-1 Kitasato, Minami-ku, Sagamihara, Kanagawa 252-0374, Japan; Department of Orthopedic Surgery, Kitasato University School of Medicine, 1-15-1 Kitasato, Minami-ku, Sagamihara, Kanagawa 252-0374, Japan; Department of Orthopedic Surgery, Kitasato University School of Medicine, 1-15-1 Kitasato, Minami-ku, Sagamihara, Kanagawa 252-0374, Japan

**Keywords:** Ellis-van Creveld syndrome, severe valgus knee deformity, adulthood

## Abstract

Ellis-van Creveld syndrome (EVC) is a rare disorder with marked valgus knee deformity, and orthopedic surgeons may experience challenges with lower limb treatment. Most previous reports have focused on EVC in childhood and few on its treatment in adulthood. Our patient was a 23-year-old woman with bilateral knee pain and gait abnormalities, with no history of orthopedic treatment. Valgus knee deformities with anterolateral depression of the lateral tibial plateaus and external rotation deformities of the lower legs were observed on radiography. We performed extra-articular osteotomy of the femurs and tibias and soft tissue release. Although the correction of each femur and tibia was good, mild valgus deformity of the lower limbs remained. This may be because the depression of the lateral tibial plateau was not repaired, and no postoperative remodeling was performed. However, 10 years post-surgery, no recurrence of the deformity was observed and walking was stable without pain.

## Introduction

Ellis-van Creveld syndrome (EVC) is an uncommon genetic disorder that was first reported by Van Ellis and Creveld in 1940. EVC has four main features: chondrodysplasia, polydactyly, congenital heart disease, and ectodermal dysplasia [1]. For orthopedic surgeons, patellar dislocation and severe valgus knee deformity have been the main problems reported in the past. However, most reports have focused on treatment in childhood [2–6], and few exist on lower limb deformity associated with EVC in adulthood [7]. We describe a case of lower limb deformity associated with EVC that was untreated in childhood and treated surgically only in adulthood.

## Case report

A 23-year-old woman presented with bilateral knee pain and gait abnormalities owing to marked lower extremity deformity. She was diagnosed with EVC as a child but had never been followed up by orthopedics because she was not in pain. On physical examination, marked valgus instability was observed in both knees, and the motion of both knees ranged from 10° to 135°. Radiographic examinations of both knees, including computed tomography, showed valgus deformity, lateral and anterior depression of the proximal lateral tibial plateau, and external rotation deformity of the lower leg. Patellar dislocation was observed in the right knee (Fig. 1). The radiological angles are presented in Table 1. The right knee underwent surgery first, followed by the left 10 months later. Lateral and medial skin incisions were made. Extensive lateral retinacular release, fractional lengthening of the distal lateral hamstring, and Z-lengthening of the iliotibial band were then performed. At this stage, peroneal nerve strain was identified, and decompression of the peroneal nerve was performed. Osteotomy was subsequently performed on the tibia. Closed-wedge varus and derotational osteotomy of the proximal tibia and open-wedge varus osteotomy of the distal femoral varus were performed. In addition, vastus medialis advancement was performed on the right knee, and patellar realignment was achieved in extension (Fig. 2). However, the knee showed a subluxation tendency owing to flexion of >30°; therefore, the postoperative rehabilitation plan progressed more slowly than that of the left knee. Range of motion training began on the left knee immediately after surgery without any restrictions, but on the right knee only after 3 weeks of postoperative immobilization in the extended position. However, ambulation exercise was performed on both knees with no loading for 3 weeks, then partial loading was initiated, and full loading was permitted at 8 weeks postoperatively. Early postoperative standing radiography showed residual mild valgus deformity of the lower limbs (Fig. 2, Table 2). In addition, the right knee showed patellar re-dislocation 2 months postoperatively, and the patellar deformity gradually progressed owing to the femoral implant; therefore, the patient underwent implant removal, tibial tubercle transfer, and medial patellofemoral ligament reconstruction 1.5 years after the initial surgery (Fig. 3). Six months later, she fell while walking and developed a fracture at the osteotomy of the right femur; open reduction and internal fixation were performed (Fig. 4). The remaining years passed uneventfully, and 10 years after the initial surgery, the patient still had mild valgus deformity of the lower limbs, but had no recurrence other than that experienced immediately after surgery, no complaints of knee pain, and a stable gait (Fig. 5, Table 2).

**Figure 1:**
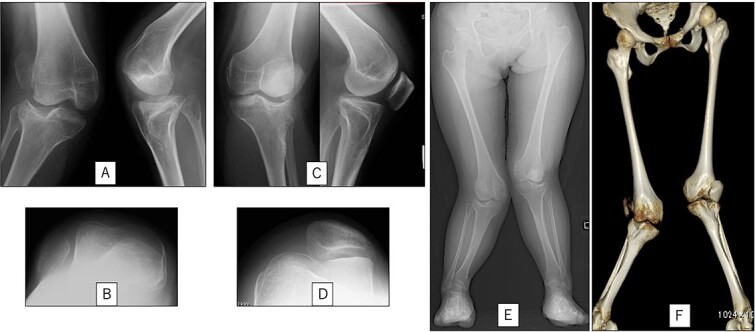
Preoperative radiography and computed tomography: (A) anteroposterior and lateral views of the right knee, (B) skyline view of the right knee, (C) anteroposterior and lateral views of the left knee, (D) skyline view of the left knee, (E) standing anteroposterior view, and (F) 3D image of the entire lower limb.

**Table 1:** Preoperative radiological angles.

	Right knee	Left knee
Femoro-tibial angle	142.0°	152.0°
Mechanical-lateral distal femoral angle	76.9°	81.6°
Medial proximal tibia angle	103.1°	102.2°

**Figure 2:**
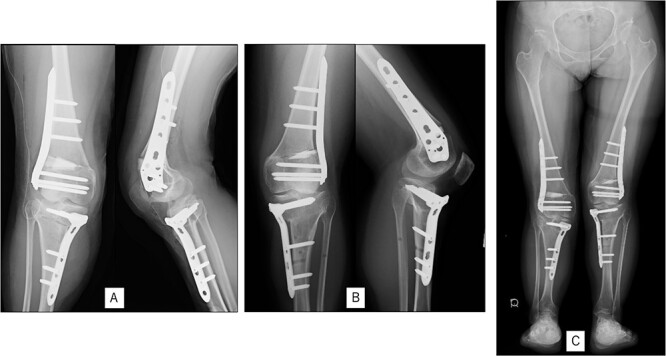
Postoperative radiography: (A) immediate postoperative radiograph of the right knee, (B) immediate postoperative radiograph of the left knee, (C) the entire lower limbs at 2 months after left knee surgery. Mild valgus deformity of the lower limbs persisted.

**Table 2:** Postoperative radiological angles.

	Right knee		Left knee	
	Early PO period	Final follow up	Early PO period	Final follow up
Femoro-tibial angle	164.6°	166.3°	164.7°	164.8°
Mechanical-lateral distal femoral angle	87.2°	89.2°	84.5°	86.1°
Medial proximal tibia angle	88.7°	88.2°	88.0°	88.3°

PO: postoperative

**Figure 3:**
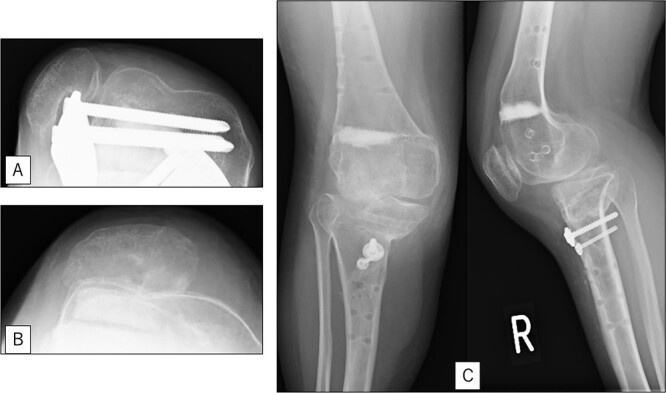
Radiography after right patellar re-dislocation and repair surgery: (A) the patella is eroded owing to contact with the femoral plate. (B) Skyline view after patellar repair surgery. (C) Anteroposterior and lateral view after patellar repair surgery.

**Figure 4:**
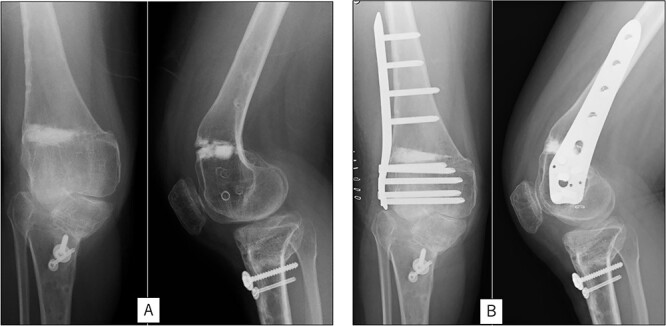
Radiography after fracture at the osteotomy of the right femur. (A) anteroposterior and lateral view immediately after fracture. (B) After open reduction and internal fixation.

**Figure 5:**
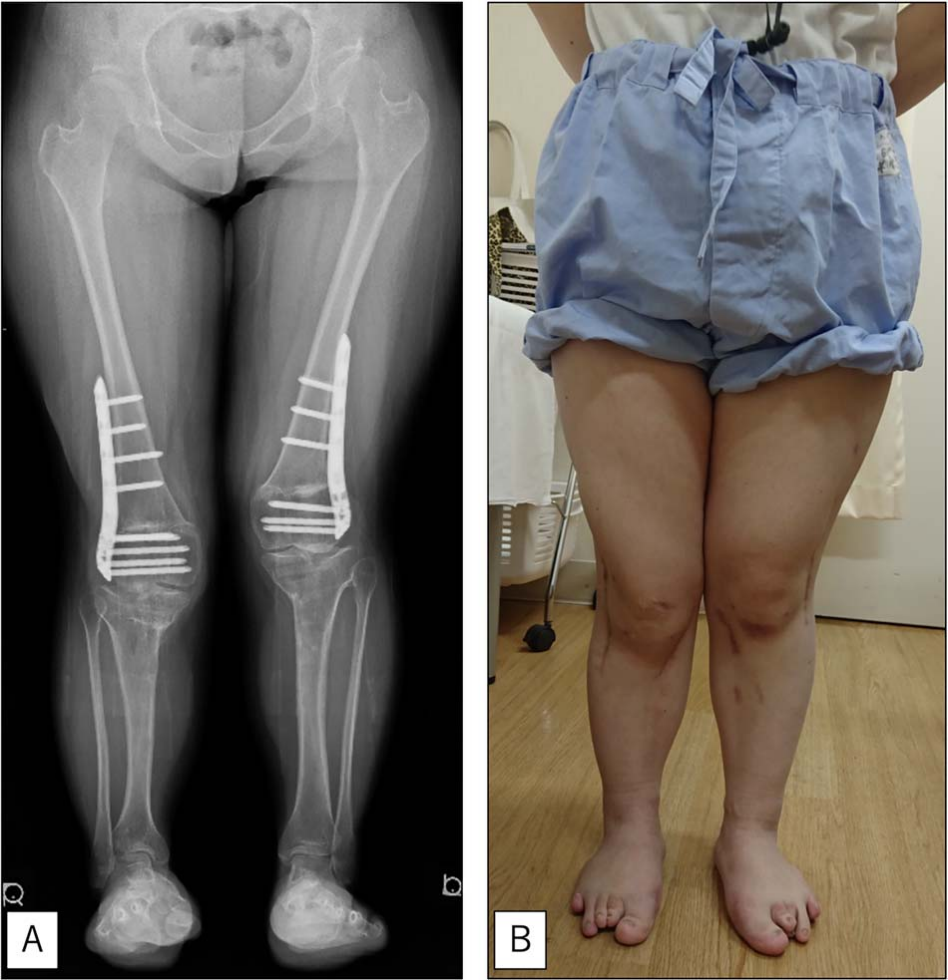
At the final follow-up 10 years after the initial surgery. (A) Standing anteroposterior view. Although mild valgus deformity of the lower limbs remained, no recurrence occurred except for that experienced immediately after surgery. (B) Photograph of the entire lower legs.

## Discussion

EVC is a rare disorder involving a severe, relentlessly progressive valgus deformity of the knee. Owing to the progressive nature of the disease, recurrence is frequent and treatment is often difficult. Although there have been several previous treatment reports, few have described treatment in adulthood (only one patient among 13 reported by Weiner *et al.* [7]).

Regarding the surgical technique, because only tibial osteotomy or inadequate soft tissue release (STR) results in recurrence, osteotomies of the femur and tibia and thorough STR are usually recommended [2, 4, 5, 7]. Weiner *et al.* [7] reported on 23 knees in 13 patients; they experienced reoperation in 2 patients (3 knees) but described favorable results with the above procedures. Several reports have also described additional procedures for patellar dislocation, such as repair and patellar tendon transfer, if necessary [2, 4, 7]. In the right knee of our patient, patellar tendon transfer was not performed because the knee was repositioned in extension using vastus medialis advancement, but it eventually dislocated again and required reoperation.

Although Palay *et al.* [8] and Feldman *et al.* [9] have reported good results with intra-articular osteotomies for significant external knee deformities with instability, Kamada *et al.* [3] have presented the only report of intra-articular osteotomy associated with EVC. Shibata *et al.* reported that the severe defect of the lateral tibial plateau was the main characteristic of valgus deformity in this syndrome and that intra-articular osteotomy should be performed for elevation of the lateral tibial plateau and bone grafting from the outset [10]. In the current patient, an extra-articular osteotomy was performed. Although the corrective angulation of the femur and tibia was effective, the depression of the lateral tibial plateau was not repaired, and no remodeling was performed postoperatively. We considered this a possible reason for the mild residual lower-limb valgus deformity. Because the growth plate was also closed in this patient and there was no associated risk of growth disturbance, intra-articular osteotomy was considered a useful method.

In the current patient, mild valgus deformity of the lower limbs persisted immediately after surgery, and there was no repair of the lateral plateau; however, the deformity had not recurred 10 years after surgery. One possible reason for the lack of recurrence is that the patient was an adult and the closure of the growth plate had been recognized preoperatively.

## Data Availability

Datasets supporting the conclusions of this study are included in this article. The corresponding author can provide the raw data upon request.
